# Supportive care needs of patients with rare chronic diseases: multi-method, cross-sectional study

**DOI:** 10.1186/s13023-020-01660-w

**Published:** 2021-01-22

**Authors:** Miram K. Depping, Natalie Uhlenbusch, Yskert von Kodolitsch, Hans F. E. Klose, Victor-Felix Mautner, Bernd Löwe

**Affiliations:** 1 Department of Psychosomatic Medicine and Psychotherapy, University Medical Center Hamburg-Eppendorf, Martinistr. 52, 20251 Hamburg, Germany; 2 Department of Cardiology, University Medical Center Hamburg-Eppendorf, Hamburg, Germany; 3 Center for Oncology, Medical Clinic and Policlinic, University Medical Center Hamburg-Eppendorf, Hamburg, Germany; 4 Clinic and Policlinic for Neurology, University Medical Center Hamburg-Eppendorf, Hamburg, Germany

**Keywords:** Supportive care needs, Psychosocial support, Rare diseases, Patient preferences, Patient reported outcomes

## Abstract

**Background:**

In the absence of a cure for the majority of rare diseases, the disease management aims to provide optimal supportive care. The goal of this study was to assess supportive care needs in patients with chronic rare diseases.

**Methods:**

Cross-sectional mixed-method study was conducted using validated self-report scales and open-ended questions to assess supportive care needs. Participants affected by rare diseases across Germany were contacted via patient organizations and centers for rare diseases. N = 304 participants with 81 different rare diseases completed the study, 81.6% were female, mean age was 44.2 years (*SD* = 12.8, range 16–74). The quantitative results regarding supportive care needs were compared to a reference population of patients affected by cancer (N = 888). Main outcomes were unmet supportive care needs of patients with rare diseases, as assessed by the Supportive Care Needs Survey (SNCS-SF34) and an open-ended question on support wishes.

**Results:**

Patients with rare diseases did not feel sufficiently supported with regard to psychological support, health system and information, physical and daily living, patient care and support, and sexuality needs. The unmet supportive care needs were significantly higher in the patient sample with rare diseases compared to the SCNS-SF34 reference sample of patients with cancer. 60% of patients with rare diseases did not feel sufficiently socially supported.

**Conclusions:**

Patients affected by rare diseases have high unmet support needs in all areas studied. Multidisciplinary care, including psychological support and the provision of information regarding the healthcare system, treatment options, disease course and sexuality, might help address these needs.

## Background

300 million people are living with a rare disease worldwide, while every single condition is defined by a prevalence of 5:10,000 [1]. This large group of patients is affected by different somatic conditions, which impact everyday life of patients in various ways. According to a European-wide survey, 8 in 10 patients and informal carers have difficulties completing daily tasks like household chores [2]. Compared to the general population, people living with a rare disease and carers report three times more often to be unhappy and depressed. 7 in 10 patients and carers of patients affected by a rare condition reduced or stopped professional activity due to their family member’s rare disease. These survey data show that patients with rare disease face high burden and that healthcare to date does not adequately address the needs of patients with rare diseases, yet. This survey had been carried out by EURORDIS-Rare Diseases Europe (European Organization for Rare Diseases, a non-governmental patient-driven alliance of patient organizations) in want for research of the lives of patients with rare diseases. A review that synthesizes qualitative studies on specific rare conditions, concludes that patients with different rare diseases all face universal burden, such as lack of information [3]. No quantitative studies to date have used validated instruments to assess unmet supportive care needs in different domains across a population of people living with different rare diseases.

Given that there is no cure for the majority of rare diseases, the management of rare diseases aims at providing optimal supportive care. Experiencing burden does not necessitate a want for help, therefore directly measuring patients’ own perceptions of their need for help may be most adequate [4]. The current study sets out to understand what patients with rare diseases wish for above the currently available forms of care. It is the first study aiming at investigating multi-domain supportive care needs in patients with different rare chronic diseases; that is whether they currently feel sufficiently supported with respect to health system and information, physical needs, patient care, sexuality and psychological needs. Supportive care needs in all 5 domains are relevant to a patient’s psychosocial well-being [4]. In order to be able to contextualize the results, we included a comparative sample of patients with other chronic conditions in the analyses, namely patients with cancer. We further explored whether this patient population feels sufficiently socially supported. Finally, we asked an open ended question to specify patients’ supportive care needs by asking which form of support they wish for.

## Method

### Participants

We aimed at recruiting *N* = 300 patients. Inclusion criteria were an age of at least 16 years, the diagnosis of any rare disease, sufficient German skills, internet access and online informed consent. We excluded patients with non-rare diseases or a diagnosis that was unclear or not confirmed by a physician. Rarity was defined based on the EURORDIS definition of rare diseases (< 1:2000) [1]. If the prevalence was unavailable, a disease was included if listed in the orphan.net register of rare diseases. We further excluded participants who completed less than 85% of the survey.

We recruited between February and July 2016 via outpatient clinics, patient organizations and self-help groups for rare diseases across Germany by distributing flyers and announcing the study online and in two patient journals. Contact information of research group members was stated. After giving consent, participants anonymously completed an online questionnaire. Ethics approval was given through the ethics committee of the Hamburg Medical Counsel on February 2, 2016 (reference number PV5088).

### Study design

This cross-sectional online study is part of the project ‘Patients for patients: qualified peer counselling and self-management for patients with rare chronic diseases’. We further investigated depression and anxiety in the sample [5].

### Measures

#### Demographic and health-related characteristics

We assessed demographic data and a variety of health-related variables. These included the diagnosis, if any family member is diagnosed with the same disease, visibility of the symptoms, comorbid diseases, current or past psychotherapeutic treatment, number of days patients have been unable to perform normal daily activities and number of doctoral visits in the past four weeks. We further asked patients if they feel sufficiently socially supported using the self-developed Item “Do you feel that you have sufficient social support?” with dichotomous response format (“yes” vs. “no”). To explore psychosocial support, we asked with whom they have supporting conversations on a regular basis. To this end, we asked the self-developed item “With whom do you have supporting conversations on a regular basis?” with the response options “nobody”, “family doctor”, “specialist physician”, “psychotherapist”, “support group”, “pastor”, “partner”, “family member”, “someone else, namely:”. All demographic and disease-related characteristics were self-reported.

#### Supportive care needs

We used the German short version of the Supportive Care Needs Survey (SCNS-SF34*;* [4]) to assess psychosocial support needs from patients’ perspective. While the instrument was developed and validated for patients with cancer [6], it has been employed investigating other patient populations [7]. The 34-item instrument measures five domains of needs (psychological, health system and information, physical and daily living, patient care and support, and sexuality needs) on a five-point response scale (1 = no need, not applicable; 2 = no need, need satisfied; 3 = low need; 4 = moderate need; 5 = high need). The *psychological* domain assesses needs related to emotions and coping, like dealing with anxiety. The *health system and information* domain assesses needs related to the treatment center and need for information about the disease, diagnosis, treatment and follow-up. *Physical and daily living* needs pertain to coping with physical symptoms, side effects of treatment and performing usual tasks and activities. The *patient care and support* domain assesses needs related to healthcare providers showing sensitivity to physical and emotional needs, privacy and choice. The subscale *sexuality needs* assesses needs related to sexual relationships.

For each domain, a standardized sum score with values ranging from 0 to 100 can be calculated with higher scores representing higher levels of need [4]. All subscales show high internal consistency with Cronbach’s alpha ranging from 0.86 to 0.96. The instrument further has demonstrated convergent validity (r = 0.48–0.56) [4].

#### Open format question

We assessed burden and support needs qualitatively by asking open format questions. To assess supportive care needs, we asked “If you could wish for some form of support, what would it be?”. In the present manuscript, we present qualitative analyses of the responses to this question.

### Data analyses

We excluded patients for whom 15% or more data were missing. We calculated means and standard deviations for all metric variables as well as frequencies for all categorical variables. For categorical data, we additionally calculated 95% confidence intervals. We calculated Cronbach’s Alpha in order to determine internal consistencies of all scales and subscales used in the study. T-tests for one sample were used to compare SCNS-SF34 means in our sample with the means of a reference sample of patients with cancer, provided in the SCNS-SF34 validation study [6] based on the transformed sum scores as described above. All statistical analyses were performed using IBM SPSS 23.

Three team members evaluated answers to the open format question using qualitative content analysis [8]. We inductively derived categories from the expressed support needs. All three team members read the answers several times. One of the researchers (NU) suggested categories, which were revised by a second researcher. Based on the feedback, first drafts of the category systems were developed. The resulting first drafts of the category system were discussed with a third researcher (AW) until reaching agreement. The final category system was then used to deductively assign open format answers to categories by two researchers independently (NU, AW). The two coders compared their results and discussed discrepancies until reaching agreement. In unclear cases, a third researcher (MKD) was consulted. We used MAXQDA to support the coding process.

## Results

### Sample

**Fig. 1:**
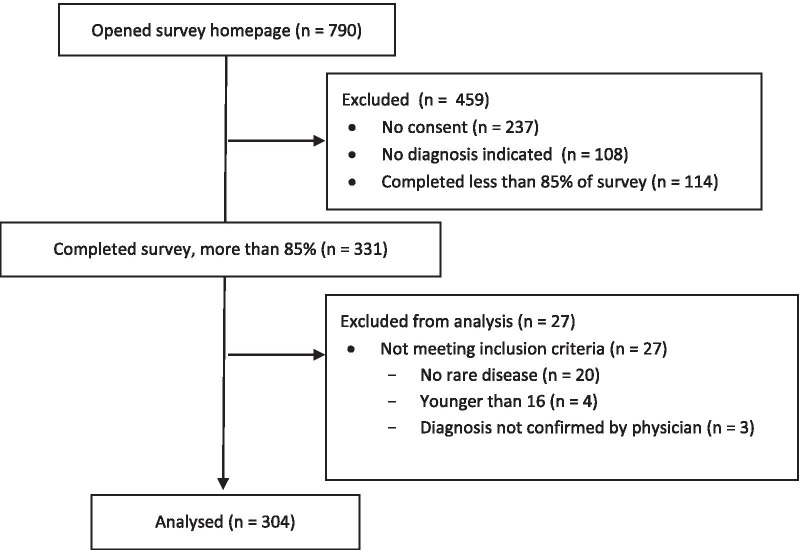
Flow diagram

N = 304 participants with 81 rare diagnoses were included in the analysis (see Fig. 1 for flow diagram). Demographic and clinical participant characteristics are displayed in Table 1 (see Appendix Table 1, online supplementary for a list of all diagnoses). 90.5% of all participants answered the open ended question.

**Table 1:** Sociodemographic and disease-related characteristics of sample including N = 304 patients with rare diseases (M_age_ = 44.2, SD = 12.8, range 16–74)

Variable		Absolute frequency	Percentage (95% CI)
*Gender*			
Female		248	81.6 (77.2, 86.0)
Male		56	18.4 (14.0, 28.8)
*Nationality*			
German		289	95.1 (92.7, 97.5)
Other		15	4.9 (2.5, 7.3)
*Relationship status*			
Single		105	34.5 (29.2, 39.8)
Married		139	45.7 (40.1, 51.3)
Separated		11	3.6 (1.5, 5.7)
Divorced		37	12.2 (8.5, 15.9)
Widowed		3	1.0 (0.1, 2.1)
Other		9	3.0 (1.1, 4.9)
*Living situation*			
Alone		70	23.0 (18.3, 27.7)
With partner		110	36.2 (30.8, 41.6)
Alone with children		18	5.9 (3.3, 8.6)
With partner and children		64	21.1 (16.5, 25.7)
With parents		24	7.9 (4.9, 10.9)
In institution		1	0.3 (0.3, 0.9)
Other		17	5.6 (3.0, 8.2)
*Professional status*			
Self-employed		13	4.3 (2.0, 6.6)
Civil servant		6	2.0 (0.4, 3.6)
Employed		121	39.8 (34.3, 45.3)
Househusband/-wife		14	4.6 (2.3, 7.0)
Seeking work		17	5.6 (3.0, 8.2)
Pension (early,-age,-widow-)		26	8.6 (5.5, 11.8)
PENSION (occupational disability/disability)		54	17.8 (13.5, 22.1)
Student		25	8.2 (5.1, 11.3)
Maternity/parental leave		2	0.7 (0.2, 1.6)
Currently on sick leave		4	1.3 (0.0, 2.6)
Other		22	7.3 (4.4, 10.2)
*Employment status*			
Full-time		120	39.5 (34.0, 45.0)
Part-time (80–99%)		20	6.6 (3.8, 3.4)
Part-time (60–79%)		21	6.9 (4.1, 9.8)
Part-time (40–59%)		18	5.9 (3.3, 8.6)
Part-time (< 40%)		18	5.9 (3.3, 8.6)
Other		107	35.2 (29.8, 40.6)
Family members diagnosed with the same illness			
		62	20.4 (15.9, 24.9)
Symptoms of disease visible			
		133	43.8 (38.2, 49.4)
At least one self-reported comorbid disease			
		209	68.8 (63.6, 74.0)
*Psychotherapeutic treatment*			
Ever		142	46.7 (41.1, 52.3)
In the past		101	33.2 (27.9, 38.5)
Currently		61	20.1 (15.6, 24.6)

	M	SD	Range
How many days have you been unable to do your job (household, school, everyday life) in the past two weeks due to illness?	2.48	4.15	0–14
Number of consultations of a doctor in last four weeks	1.96	1.81	0–12

### Social support

The majority of all participants reported not to feel sufficiently socially supported (see Table 2). Patients sought support from personal contacts, including partner, family members and self-help groups, rather than medical professionals (see Table 2). Patients report less support with increasing specialization of the medical professionals.

**Table 2:** Social support of sample including N = 304 Patients with rare diseases

Variable		Absolute frequency	Percentage (95% CI)
Do you feel that you have sufficient social support?	Yes	122	40.1 (34.6, 45.6)
	No	182	59.9 (54.4, 65.4)
With whom do you have supporting conversations on a regular basis?			
Nobody	Yes	69	22.7 (18.0, 27.4)
	No	235	77.3 (72.6, 82.0)
General practitioner	Yes	31	10.2 (6.8, 13.6)
	No	273	89.8 (86.4, 93.2)
Specialist physician	Yes	27	8.9 (5.7, 12.1)
	No	277	91.1 (87.9, 94.3)
Psychotherapist	Yes	51	16.8 (12.6, 21.0)
	No	253	83.2 (79.0, 87.4)
Self-help group	Yes	55	18.1 (13.8, 22.4)
	No	249	81.9 (77.6, 86.2)
Pastor	Yes	6	2.0 (0.4, 3.6)
	No	298	98.0 (96.4, 99.6)
Partner	Yes	107	35.2 (29.8, 40.6)
	No	197	64.8 (59.4, 70.2)
Family member	Yes	97	31.9 (26.7, 37.1)
	No	207	68.1 (62.9, 73.3)

### Supportive care needs

**Fig. 2:**
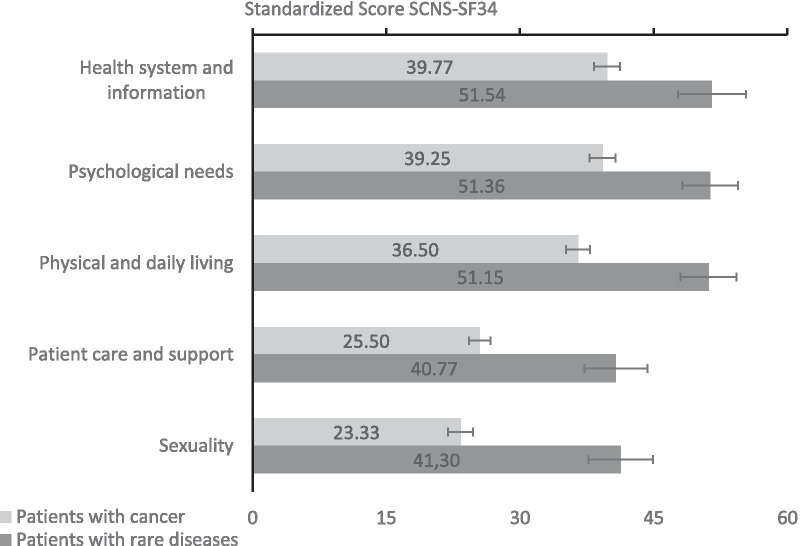
Unmet supportive care needs of patients with rare diseases (N = 304) in comparison with SCNS reference sample of patients with cancer (N = 888) domains. Error bars indicate 95% CI

Standardized sum scores of the SCNS-SF34 subscales were compared to the mean values of a reference sample affected by cancer (see Fig. 2). Patients with rare diseases reported higher supportive care needs than the reference sample with respect to psychological support needs (*t*(283) = 7.67, *p* < 0.001 Cohen’s *d* = 0.43), support needs regarding health system and information (*t*(283) = 6.04, *p* < 0.001, Cohen’s *d* = 0.40), physical and daily living support needs (*t*(279) = 9.15, *p* < 0.001, Cohen’s *d* = 0.54), supportive care needs for patient care and support (*t*(294) = 8.47, *p* < 0.001, Cohen’s *d* = 0.60) and support needs concerning sexuality (*t*(293) = 9.80, *p* < 0.001, Cohen’s *d* = 0.64). Overall, patients with rare disease in the current sample wished for more supportive care in all domains and their reported care needs succeeded that of the reference sample in all domains.

### Supportive care needs—qualitative analysis

For elaboration, we analyzed the open-ended question. Table 3 presents the supportive care needs patients named by the subdomains listed above. In addition to these domains, patients further named the wish to be cured from their disease or reported that they currently had sufficient support. Participants asked for more and “better” information on the disease for themselves, their spouses, carers and the general public.

**Table 3:** Support wishes according to open format answers (N = 275)

Wishes regarding…		Total number of mentions
*Health system and information needs*		
Healthcare and information		277
Facilitated access to experts and treatment		49
Information transfer for the patients		28
More support by funding agencies		27
Psychotherapy		23
Better informed physicians		23
Adequate and best possible medical care		21
Information transfer for others		13
Improved/ facilitated cooperation between different healthcare providers		13
Assistance in bureaucratic procedures		11
Guidance and consultation		9
Rehabilitation interventions		6
More research		6
Pain therapy		4
Physiotherapy		4
Support for relatives		3
Alternative treatments		2
Other treatment measures		2
Euthanasia		2
*Patient care and support needs*		
More personable physicians		28
Being more involved in treatment decisions		3
*Psychological Needs*		
Coping strategies		4
Learning disease management strategies		3
Learning emotion regulation strategies		1
Social/emotional support		77
Inclusion/more tolerance and acceptance in society		31
Social and emotional support		22
Contact to peers with the same disease/ self-help		20
Social belonging/ participation		4
*Physical and daily living needs*		
Support in daily life		53
Support for everyday life tasks		25
Job-related facilitations		12
Assistive technology		11
Increased disease-specific leisure opportunities		5
*Other*		
A symptom-free status/ cure		8
No specific support wishes		29
Uncertainty about support wishes		12
Sufficient support available		12
Unspecific support wishes		5

Better education of others who do not have the disease that they can better understand me and not label one as handicapped.

In addition to the need for information, participants reported that they wish for better access to self-help and contact to other patients affected by the same condition:

After diagnosis, to get better information of the physicians and an internet address of a patient forum of the illness, perhaps a telephone number or e-mail address of someone also ill from close by for exchange.

Contact to other patients can be seen both as a source of information as well as addressing social needs of belonging. Moreover, patients named a series of different specific types of care that they would need, like physiotherapy or support for their family members. They also wished for a long-term supportive contact through psychotherapy that would go beyond standard contingent of therapy normally provided and covered by insurance providers.

## Conclusion

This is the first study to quantitatively and qualitatively assess supportive care needs among patients affected by rare chronic conditions. This study shows that patients with different rare diseases have high unmet support needs on different domains.

Participants reported unmet *psychological needs*. Qualitative analysis further revealed that, psychological needs pertain to help in developing *individual strategies* like disease management strategies and emotion regulation strategies. Participants reported unmet social needs in their intimate relationships. Consistent with previous research [9], participants expressed the need to be in contact with other patients with the same condition. In the present study, participants also expressed a desire for more acceptance on a societal level. This desire may stem from the experience of stigmatization. Stigma was identified as a shared challenge in a large sample of patients affected by rare diseases [10]. In a previous interview study, patients with the rare diagnosis sarcoidosis, as well as their partners, consistently reported feeling regularly misunderstood because of the general unawareness of the condition [11].

Unmet needs were further reported in the *health system and information* domain. Qualitative analysis further revealed that participants wished for facilitated access to experts and treatment, as well as more information about their condition that is tailored to them to aid their understanding of it. Furthermore, participants wished for additional, specific healthcare services like physiotherapy or (extended quotas of) psychotherapy. These findings are in line with a previous qualitative study, which identified topics that patients with diverging rare diagnoses wished for information about, such as the impact of the disease on their daily activities. Patients in this study also wished for the information to be presented in a clear way, without foreign words that make it difficult for them to understand [9].

In the realm of *physical and daily living*, participants wished for more support*.* Responding to the open-ended question, participants also named the need for support in everyday life tasks and additionally for job-related facilitations (e.g. flexible time allocation in order to be able to attend medical appointments during work hours).

Unmet needs were reported for *patient care and support*. Qualitative analysis of patients’ responses showed that they wish for more sensitive communication with physicians, in particular.

Participants reported to wish for more support than they currently receive in sexual relationships in the questionnaire (*sexuality needs domain*), e.g. by receiving information on sexual relationships. In response to the open-ended question about support wishes, no participant named support needs with respect to sexuality. This may be because participants felt ashamed to raise these issues. Alternatively, given the open-ended format, they may have deemed the reporting of other themes more pressing. In a scoping review on patients’ supportive needs in living with ALS; loss of sexuality and intimacy was also identified as an area patients wanted help with [12].

This was the first mixed-method study to investigate supportive care needs across different diagnoses of rare conditions from the perspective of patients. A trans-diagnostic approach allows for the identification of a scope for disease management and effective care tailored to the shared needs of a large group of patients. Previous research has primarily focused on single conditions (e.g. sarcoidosis [11]. The focus on care needs complements an emerging field of research investigating challenges faced by patients with rare diseases [10, 13]. In the face of finite healthcare resources, addressing needs of patients with rare diseases can help provide better care for a group of patients for whom healthcare professionals are otherwise confronted with the lack of a cure and knowledge about specific conditions and their management. Understanding the shared supportive care needs of groups of patients with rare diseases can therefore guide healthcare professionals in advising and treating patients with rare diseases who often present as “one of their kind” in primary care. Overall, the results of studies on single conditions are in line with the current study, despite previous studies focusing on single conditions and employing different methods for the assessment. This supports the notion that patients with different rare diseases may share generic burden and supportive care needs. Further studies on the shared needs of patients with different rare diseases qualitatively assessed the perspectives of experts on needs in the process that leads up to the diagnosis [14] or studied psychosocial supportive needs of families and care-givers [15]. The present research extends these perspectives.

A weakness lies in the sampling; it does not reflect a representative picture of patients with rare diseases. We recruited via outpatient clinics, patient organizations and self-help groups for rare diseases. It is likely that we primarily reached patients who, it can be presumed, are well integrated into care structures. Patients with rare diseases for whom there are no patient associations or specialist clinics available may have even greater support needs. More women than men participated, potentially because of their interest in the topic, which might be a source of bias. This gender ratio is similar to that of a previous study in this field on information needs [9].

### Implications and outlook: how can we improve care for patients with rare diseases?

Assessing supportive care needs enables service providers to identify gaps in existing services and prioritize resource allocation to those aspects of care that need improvement [16]. The current study shows that adult patients affected by rare diseases need supportive care in a broad range of domains. Quality of life could be improved by implementing interventions on different levels of a patient’s ecosystem: The individual level (e.g. psychological), the patient’s microsystem (e.g. social and work environment or contact with physicians) as well as higher order levels, i.e. the healthcare system or the general public.

Regarding the latter, patients report the need for multidisciplinary care, which could be implemented in centers for rare diseases and that in part could be coordinated through general practitioners in the absence thereof. On the level of health policy, assumption of costs for specific healthcare services, like extended quotas of psychotherapy could be reviewed. To further improve care for patients with rare diseases, access to experts and treatment could be facilitated by implementing tele medical measures with specialized physicians and psychotherapists. Finally, patients want more awareness of their diseases and less stigmatization, which could be achieved by information campaigns.

In the patient’s microsystem, healthcare professionals can systematically inquire needs along the dimensions reported above: whether patients feel sufficiently supported with respect to psychological needs, health system and information, physical needs, patient care, and sexuality. Based on this assessment, individual assistive measures could be taken and other providers integrated in collaborative care. Screening instruments could account for support needs and mental health, e.g. SCNS-SF34 [4] for supportive care needs, PHQ-9 for depression [17], GAD-7 for anxiety [18, 19] and SSD-12 for distressing symptom burden [20]. Furthermore, the provision of comprehensible information in written or verbatim delivery could help address patients’ information needs. For this purpose, checklists could help healthcare professionals to ensure that they have covered important issues. One way to address the need to be in contact with other patients with the same condition could be to involve expert patients in multidisciplinary care. On an individual level, implications for care can be to provide sufficient psychological aid to individually address psychological needs.

This study shows that patients with different rare diseases have high unmet support needs on different domains. Interventions addressing these needs can help to provide better and more comprehensive care. Future research could further differentiate shared needs from those specific to single conditions in order to optimize treatment.

## Data Availability

Data cannot be made publicly available due to ethical reasons. In the area of rare diseases, information about the diagnosis in combination with personal information may compromise anonymity and confidentiality of the participants. The independent ethics committee of the Hamburg Medical Chamber assessed our research project beforehand. The ethics vote allows sharing data with eligible researchers but we do not have approval to share the data publicly. Researchers interested in getting access to the data should feel free to contact the corresponding author (Miriam K. Depping—m.depping@uke.de) or the principal investigator (Prof. Dr. Bernd Löwe—b.loewe@uke.de). The ethics committee can be contacted at: ethik@aekhh.de.
